# Ribitol restores functionally glycosylated α-dystroglycan and improves muscle function in dystrophic *FKRP*-mutant mice

**DOI:** 10.1038/s41467-018-05990-z

**Published:** 2018-08-27

**Authors:** Marcela P. Cataldi, Peijuan Lu, Anthony Blaeser, Qi Long Lu

**Affiliations:** McColl-Lockwood Laboratory for Muscular Dystrophy Research, Cannon Research Center, Carolinas Medical Center, Carolinas Healthcare System, Charlotte, NC 28203 USA

## Abstract

*O*-mannosylated α-dystroglycan (α-DG) serves as receptors for cell–cell and cell–extracellular matrix adhesion and signaling. Hypoglycosylation of α-DG is involved in cancer progression and underlies dystroglycanopathy with aberrant neuronal development. Here we report that ribitol, a pentose alcohol with previously unknown function in mammalian cells, partially restores functional *O*-mannosylation of α-DG (F-α-DG) in the dystroglycanopathy model containing a *P448L* mutation in *fukutin-related protein* (*FKRP*) gene, which is clinically associated with severe congenital muscular dystrophy. Oral administration of ribitol increases levels of ribitol-5-phosphate and CDP-ribitol and restores therapeutic levels of F-α-DG in skeletal and cardiac muscles. Furthermore, ribitol, given before and after the onset of disease phenotype, reduces skeletal muscle pathology, significantly decreases cardiac fibrosis and improves skeletal and respiratory functions in the FKRP mutant mice. Ribitol treatment presents a new class, low risk, and easy to administer experimental therapy to restore F-α-DG in FKRP-related muscular dystrophy.

## Abstract (web summary)

Mutations in FKRP impair glycosylation of alpha-dystroglycan, leading to muscular dystrophy. Here, the authors show that oral administration of ribitol increases dystropglycan glycosylation and ameliorates symptoms of muscular dystrophy in FKRP-deficient mouse models.

## Introduction

O-mannosylation of α-dystroglycan (α-DG), specifically the synthesis of laminin-binding matriglycan (F-α-DG), is conserved at least in vertebrates and critical for neuronal development and muscle integrity and functions^1–3^. F-α-DG also acts as viral receptors and plays prominent roles in epithelium adhesion and signaling^4^. Hypoglycosylation is involved in cancer development and progression and underlie specific types of muscular dystrophy, in particular dystroglycanopathy with and without defects in neuronal development^5–7^. One most common dystroglycanopathy caused by mutations in the *fukutin-related protein* (*FKRP*) gene manifests a wide range of disease severity from mild limb girdle muscular dystrophy (LGMD) 2I to severe congenital muscular dystrophy (CMD), Walker–Warburg syndrome, and muscle–eye–brain disease^8–10^. Lack of F-α-DG results in progressive degeneration of both skeletal and cardiac muscles. Consequently, patients gradually lose mobility with impaired and ultimately failure of respiratory and cardiac functions^11,12^. The severe forms of the disease can affect central nerve and optical systems with developmental delay and mental retardation^8,9^. Currently no treatment is available, although several experimental therapies are being tested pre-clinically^13,14^.

α-DG is a peripheral membrane protein extensively glycosylated with both *N*-linked and *O*-linked glycans, the latter acting as a cellular receptor for laminin and other extracellular matrix (ECM) proteins, including agrin, perlecan, neurexin, and pikachurin^15–20^. The interaction of α-DG with ECM proteins is critical for maintaining muscle integrity^2^. The structure of the laminin-binding *O*-mannosylated glycan on α-DG (F-α-DG) has recently been delineated with the following chain: (3GlcA-β1-3Xyl-α1) *n*-3GlcA-β1-4Xyl-Rbo5P-1Rbo5P-3GalNAc-β1-3GlcNAc-β1–4(P-6) Man-1-Thr/ser^21–23^. The glycan chain extension pathway is completed by the following proposed transferase activity: POMT1 and POMT2 catalyze the initial *O-*mannosylation of the proteins^24^. Further extension of the sugar chain is carried out by POMGnT2 (GTDC2)^25,26^, B3GALNT2^27^, FKTN, FKRP^21^, TMEM5^28^, and B4GAT1 successively^29^. Finally, LARGE (like-acetylglucosaminyltransferase) acts as a bifunctional glycosyltrasferase having both xylosyltransferase and glucuronyltransferase activities, producing repeated units of 3GlcA-1-3Xyl-1^30^.

The advances in unraveling the pathway for F-α-DG open new venues for experimental therapy. Recently, isoprenoid synthase domain containing (ISPD) has been identified as a cytidyltransferase (pyrophosphorylase) producing cytidine 5′-diphosphate (CDP)-ribitol^21–23,31^. Furthermore, CDP-ribitol has now been confirmed by several groups as the substrate of FKRP and FKTN for the extension of the glycan chain of α-DG with ribitol-5-phosphate (ribitol-5P)^23,31^. Interestingly, a study from Gerin et al.^23^ demonstrated that ribitol treatment of human embryonic kidney 293 cells overexpressing ISPD and patient-derived ISPD-deficient fibroblasts leads to an increase of CDP-ribitol levels and partially corrects the defect in F-α-DG caused by loss of ISPD function^23^. The authors also noted that overexpression of ISPD increased ribitol incorporation into α-DG in wild-type cells, suggesting that the levels of CDP-ribitol might be a limiting factor of this *O*-mannosylation. These results raise one intriguing possibility: if conversion of ribitol to CDP-ribitol is not a rate-limiting process in muscles in the *FKRP* mutant mice in vivo as suggested in the normal mice^23^, then an increase in the intracellular levels of ribitol could increase the levels of CDP-ribitol. Since most mutant FKRPs retain at least partial function^32^, an increase in the levels of CDP-ribitol substrate might enhance the efficiency of remaining function of mutant FKRP, thus compensating for the reduced function of mutant FKRPs and enhancing F-α-DG.

In this study, we test our hypothesis in the *FKRP* mutant mice containing *P448L* mutation which is associated with CMD in clinic. Our results show that ribitol treatment increases levels of ribitol-5P and CDP-ribitol in muscle tissue and can effectively restore therapeutic levels of F-α-DG both before and after the onset of the disease phenotype. This results in significant improvement in muscle pathology and functions. Moreover, no side effects are detected in histology and functions of liver and kidney, muscle development, body weight, and behavior of the animals. To the best of our knowledge, this is the first demonstration that a pentose alcohol ribitol constitutes a potentially effective and safe treatment to FKRP-related dystroglycanopathies.

## Results

### Ribitol increases F-α-DG in cardiac and skeletal muscles

**Fig. 1:**
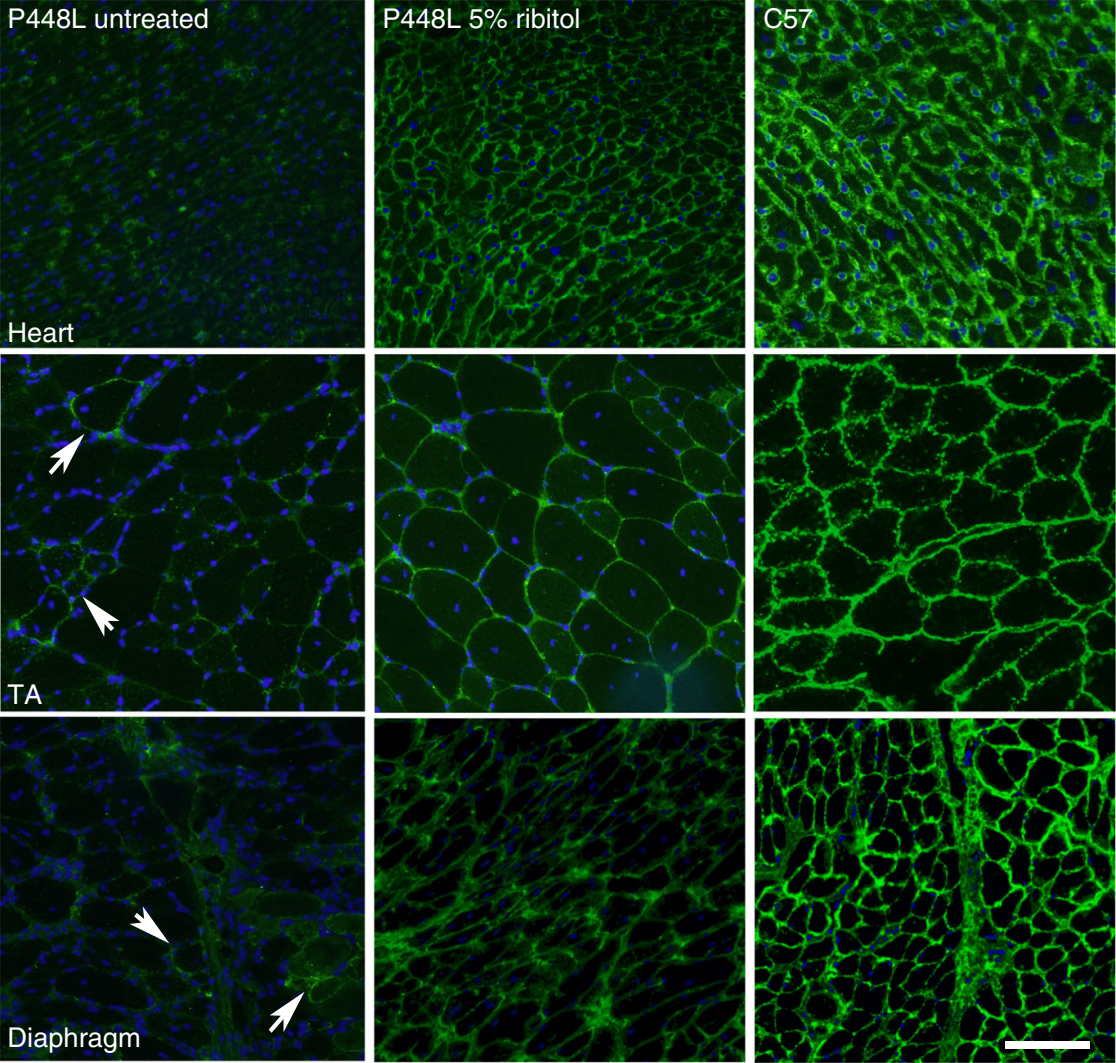
Induction of F-α-DG in cardiac and skeletal muscles in *P448L* mutant mouse treated with ribitol for 1 month. Four-week-old *P448L* mice were given drinking water only (*n* = 4), or drinking water supplemented with 5% ribitol (*n* = 4) for 1 month. Immunohistochemical staining with IIH6C4 antibody of cardiac (heart), tibialis anterior (TA), and diaphragm muscles from the untreated and 5% ribitol-treated *P448L* mice (left and middle panel, respectively), and *C57BL/6* control (C57, right panel). Arrows indicate the revertant fibers expressing detectable F-α-DG. Scale bar, 50 μm. Cellular nuclei were counterstained with DAPI (blue)

We have previously reported an FKRP mouse model containing a *P448L* mutation (P448L) with onset of the dystrophic pathology as early as 3 weeks of age^33^. In the pilot experiment, 4-week-old *P448L* mice were treated with drinking water supplemented with 5% ribitol for 1 month. Glycosylation of α-DG was analyzed by immunohistochemistry with a monoclonal antibody, IIH6C4, specifically recognizing the laminin-binding epitopes of F-α-DG. Consistent with early reports, F-α-DG was undetectable in cardiac and skeletal muscles of the untreated *P448L* mice given drinking water only, except for isolated small clusters of revertant fibers in skeletal muscles^33,34^, and one or two fibers expressing F-α-DG in cardiac muscle (Fig. 1). In contrast, oral 5% ribitol treatment visibly increased F-α-DG in the heart, diaphragm, and limb muscles. The signals of F-α-DG were consistently and clearly detected in the large proportion of diaphragm muscle fibers of the ribitol-treated mice. Interestingly, the signals for F-α-DG were easily detected with higher homogeneity in the cardiac muscle than in the skeletal muscles. Signals for F-α-DG in all the muscles of ribitol-treated mice were in general weaker when compared to the same muscle of *C57* mice.

### Ribitol increases levels of ribitol-5P and CDP-ribitol

**Fig. 2:**
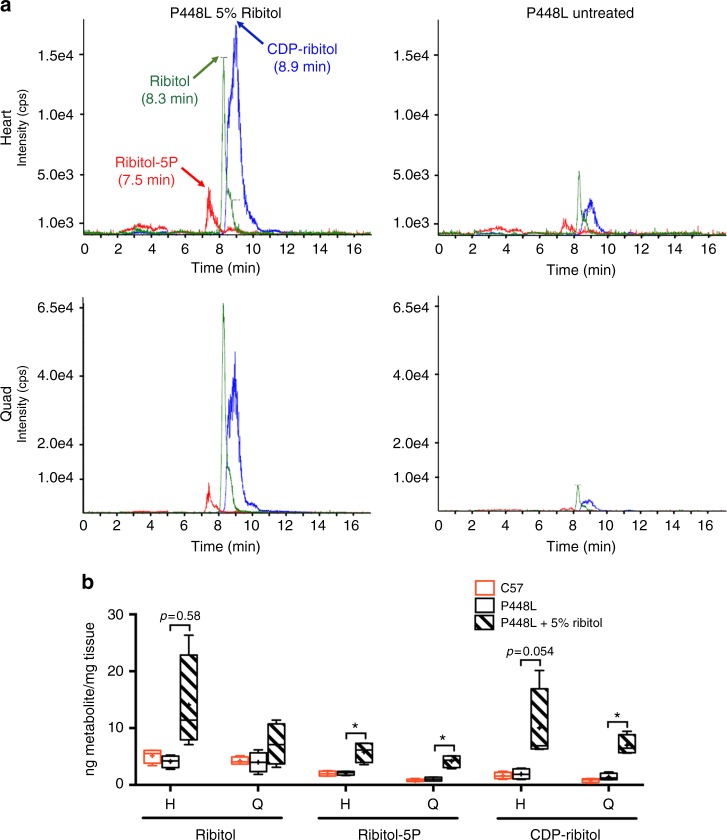
Detection and quantification of ribitol, ribitol-5P, and CDP-ribitol by LC/MS-MS. **a** LC/MS-MS detection of ribitol, ribitol-5P, and CDP-ribitol from heart and quadriceps of 4-week-old *P448L* mice treated with drinking water only (untreated) or water supplemented with 5% ribitol for 1 month. **b** Quantification of ribitol, ribitol-5P, and CDP-ribitol levels by LC/MS-MS from heart (H) and quadriceps (Q) of untreated and 5% ribitol-treated *P448L* mice, and untreated C57 control mice (*n* = 4 for all cohorts). Box represents 25th and 75th percentiles. Line represents median and “+” represents mean. Whiskers extend from min to max value. Unpaired *t* test, *p ≤ 0.05

To evaluate whether oral administration of ribitol increases levels of ribitol-5P and CDP-ribitol in cardiac and skeletal muscles of mutant mice, we analyzed and quantified ribitol, ribitol-5P and CDP-ribitol in muscle tissues by liquid chromatography with tandem mass spectrometry (LC/MS-MS). Ribitol (Sigma) as well as synthesized ribitol-5P and CDP-ribitol (Z-Biotech) were used to develop the detection method and to establish the standard curves for the quantification of the metabolites (Supplementary Figure 1a and 1b, respectively). Endogenous levels of ribitol, ribitol-5P, and CDP-ribitol were similar between untreated mutant *P448L* and *C57* control mice (Fig. 2b). The three metabolites showed increased levels in heart and quadriceps of the 5% ribitol-treated mice compared to untreated *P448L* mice (Fig. 2a, b). Levels of CDP-ribitol were at least fourfold higher in heart and quadriceps of treated mice when compared to untreated mice and the difference of ribitol-5P and CDP-ribitol levels were statistically significant in both heart and quadriceps (Fig. 2b). The levels of the metabolites were apparently higher in the heart tissues than in the skeletal muscles.

To address the question whether the orally administrated ribitol is, in fact, converted to ribitol-5P and CDP-ribitol, we treated differentiated C2C12 myotubes with isotopically labeled ^13^C_5_-ribitol (^13^C-ribitol) in vitro. ^13^C-ribitol (Omicron Biochemicals Inc.) was used to develop the LC/MS-MS method for detection of ^13^C-ribitol in cell samples (Supplementary Figure 2a). The multi-reaction monitoring methods for ^13^C-ribitol-5P and CDP-^13^C-ribitol were inferred from their non-labeled analogs (mass + 5 amu). The LC/MS-MS analysis from the untreated cells showed low levels of endogenous ribitol, ribitol-5P, and CDP-ribitol and the absence of ^13^C-labeled analogs. However, the cells treated with ^13^C-ribitol showed clearly elevated levels of ^13^C-ribitol-5P and CDP-^13^C-ribitol, as well as ^13^C-ribitol, but only background levels of endogenous analogs (ribitol, ribitol-5P, and CDP-ribitol) as detected in the untreated cells (Supplementary Figure 2b). All together, these results confirm that exogenous ribitol can be converted to ribitol-5P and most importantly CDP-ribitol, the FKRP substrate for F-α-DG synthesis.

### Long-term induction of F-α-DG by ribitol in *FKRP* mutant mice

**Fig. 3:**
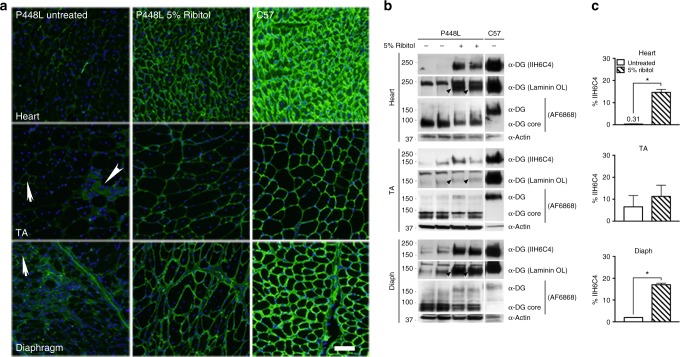
Induction of F-α-DG in cardiac and skeletal muscles of *P448L* mice treated with ribitol for 6 months. Seven-week-old *P448L* mice were given drinking water only (*n* = 4), or drinking water supplemented with 5% ribitol (*n* = 4). **a** IIH6C4 immunohistochemical staining of cardiac (heart), tibialis anterior (TA), and diaphragm tissues from either untreated or 5% ribitol-treated *P448L* mice (left and middle panel, respectively), and *C57* mice. Nuclei were counterstained with DAPI (blue). Arrows indicate the revertant fibers expressing F-α-DG. Arrowhead indicates the degenerating fibers and focal accumulation of nuclei. Scale bar, 50 μm. **b** Western blot and laminin overlay assay of lysates from heart, TA, and diaphragm (diaph) of two untreated (−) or two ribitol-treated (+) *P448L*, and *C57* mice. F-α-DG was detected by blotting with IIH6C4 and by laminin overlay assay (Laminin OL). Core of α-DG was detected by AF6868 antibody with weaker signals for the ribitol-treated samples. Detection of α-actin was used as a loading control. Arrowheads indicate laminin-binding bands. The upper band with laminin-binding assay are endogenous laminin present in all samples. **c** Quantification of IIH6C4 band intensity from western blot. Values were normalized to α-actin expression for each tissue and presented as percentage of C57 levels. Error bars represent mean ± SEM. Unpaired *t* test. *p ≤ 0.05

To assess whether ribitol treatment can maintain a long-term effect on glycosylation of α-DG in mutant mice already exhibiting severe dystrophic phenotype, we treated the *P448L* mice at the age of 7 weeks with 5% ribitol in drinking water for up to 3 and 6 months. Consistent with the 1-month treatment, all muscles from both cohorts of treated mice showed a clear increase in the levels of F-α-DG by immunofluorescence with IIH6C4 (Fig. 3a and Supplementary Figure 3a for 6-month and 3-month treatments, respectively). Nearly all fibers in the cardiac muscle, and a majority of fibers in both diaphragm and limb muscles, were positive for F-α-DG (Fig. 3a). Signal distribution and intensity for F-α-DG were generally similar in the same muscles between 3-month and 6-month ribitol-treated cohorts. The enhanced expression of F-α-DG by ribitol was further confirmed by western blot analysis with IIH6C4 antibody, reaching up to 14 and 17% of normal levels in the cardiac muscle and diaphragm, respectively (Fig. 3b, c). Enhanced expression of F-α-DG was further demonstrated by western blot with the antibody AF6868 ^35^ (Fig. 3b). Finally, functionality of the ribitol-induced glycosylated α-DG was supported by laminin overlay assay (Fig. 3b).

To evaluate whether administration of ribitol affects expression of glycosyltransferases responsible for the synthesis of Core M3 glycan on α-DG, we measured levels of mutant FKRP and LARGE transcripts by quantitative real-time PCR in cardiac muscle, limb muscle, and diaphragm (Supplementary Figure 3b). No statistically significant difference in FKRP and LARGE transcript levels was observed between treated and untreated samples in any of the tissues, suggesting that the effect of ribitol on levels of F-α-DG is independent to expression levels of the glycosyltransferases.

### 5% Ribitol improves pathology and respiratory function

**Fig. 4:**
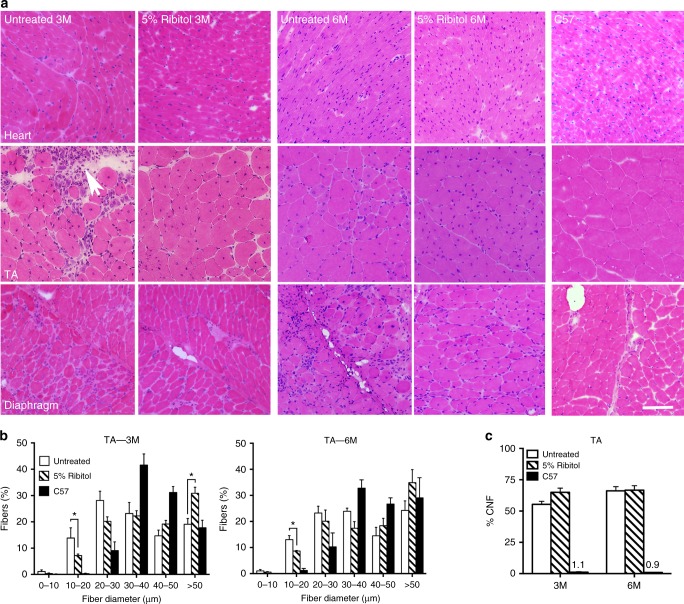
Histopathology of muscle tissues from ribitol-treated *P448L* mice. Seven-week-old *P448L* mice were given drinking water only, or drinking water supplemented with 5% ribitol for either 3 months (3M) or 6 months (6M). **a** H&E staining of heart, tibialis anterior (TA), and diaphragm tissues from untreated (untreated 3M and untreated 6M) or ribitol-treated (5% ribitol 3M and 5% ribitol 6M) *P448L* mice, and *C57* mice. Arrow indicates areas of heavy infiltration in the control TA muscle. Scale bar, 50 μm. **b** Fiber size distribution of TA muscles of either untreated (*n* = 3) or ribitol-treated (*n* = 6) *P448L* mutant mice, and *C57* (*n* = 3) mice. **c** Percentage of CNF in TA muscles of *P448L* mice treated with 5% ribitol for 3M (*n* = 6) and 6M (*n* = 4), or aged-matched untreated (*n* = 3) *P448L* mice and *C57* (*n* = 3) mice. Error bars represent mean ± SEM. Unpaired *t* test, *p ≤ 0.05

**Fig. 5:**
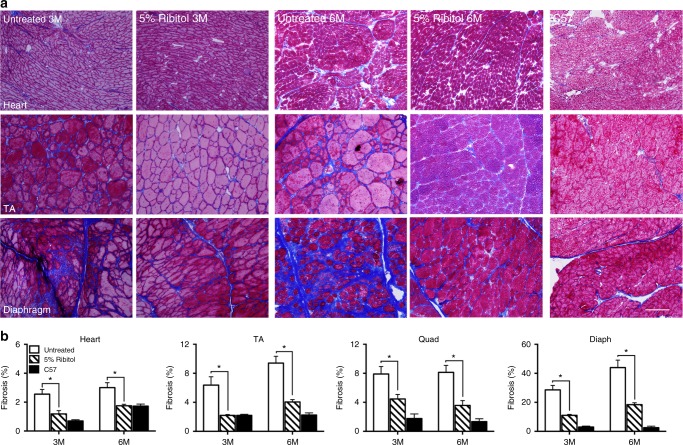
Effect of ribitol treatment on muscle fibrosis in *P448L* mice. Seven-week-old *P448L* mice were given drinking water only or drinking water supplemented with 5% ribitol for either 3 months (3M) or 6 months (6M). **a** Masson’s Trichrome staining of heart, tibialis anterior (TA), and diaphragm muscles from untreated (untreated 3M and untreated 6M) or 5% ribitol-treated (5% ribitol 3M and 5% ribitol 6M) *P448L* and *C57* mice. Blue staining represents area of fibrotic tissue. Scale bar, 100 μm. **b** Percentage of fibrotic areas quantified from Masson’s Trichrome staining of 3 months (3M) and 6 months (6M) untreated and 5% ribitol-treated heart, TA, quadriceps (QUAD), and diaphragm (DIAPH) muscles (*n* = 6 for 3M, *n* = 4 for all other cohorts). Error bars represent mean ± SEM. Unpaired *t* test, *p ≤ 0.05

Therapeutic effect of 3-month and 6-month treatments with 5% ribitol on dystrophic pathology of skeletal muscles was demonstrated by histology. Hematoxylin and eosin (H&E) staining showed the large areas of degenerating fibers, high variation in fiber sizes, and high percentage of centrally nucleated fibers (CNFs) in the skeletal muscles of the untreated *P448L* mice (Fig. 4a, Supplementary Figure 4a, and Fig. 5). This was associated with focal inflammatory infiltrates. Treatment with ribitol improved the dystrophic pathology of limb muscles as evidenced by the diminished foci of necrotic fibers and a more homogenously distributed fiber size. Quantitative analysis from TA and quadriceps showed a statistically significant decrease in the number of fibers with small diameters (newly regenerated) indicating a decrease in degeneration after both 3-month and 6-month ribitol treatments (Fig. 4b and Supplementary Figure 4b for TA and quadriceps, respectively). Furthermore, both 3-month and 6-month ribitol treatments significantly decreased areas of fibrotic tissue detected by Masson’s Trichrome staining when compared to untreated mice (Fig. 5a, b). No significant difference in percentage of CNF was observed between ribitol-treated and untreated *P448L* mice (Fig. 4c and Supplementary Figure 4c for TA and quadriceps, respectively). This is expected as significant CNF reduction could only be achieved with high dosage of viral particles with adeno-associated virus (AAV) gene therapy in the same mouse model^13,36^.

Importantly, 5% ribitol treatment significantly reduced pathology of the diaphragm. Large foci of degenerating fibers were common in the untreated diaphragms, but became rarely observed in all the mice after 3-month and 6-month ribitol treatments (Fig. 4a and Supplementary Figure 5). The most striking improvement was the degree of fibrosis. The diaphragm of the untreated mice showed heavy fibrosis at the 3-month time point (28.6% of tissue cross-section area), reaching more than 40% 6 months after the study initiation (Fig. 5a, b and Supplementary Figure 6). However, the amount of fibrotic tissues in the ribitol-treated cohorts was significantly reduced to 11 and 18% after 3-month and 6-month treatment, respectively.

The cardiac muscle of the *P448L* mice has limited pathology with only a small increase in fibrotic area as disease progresses^27,28^. H&E staining did not show infiltration and degenerating fibers in both the ribitol-treated and the ribitol-untreated mice (Fig. 4a). However, a significant reduction in fibrotic area was observed in the cardiac muscle of both 3-month and 6-month ribitol-treated groups when compared to the ribitol-untreated groups (Fig. 5a, b).

The significant improvement in histology of diaphragm with ribitol treatment was associated with improvement in respiratory function shown by whole-body plethysmography at 3 and 6 months post initiation of the treatment. A trend of improvement was observed in tidal volume (TV), expiratory volume (EV), and minute volume (MV) in both 3-month and 6-month 5% ribitol-treated groups compared to the untreated *P448L* mice (Supplementary Figure 7a). Importantly, the improvement in EV and MV after 6-month ribitol treatment became statistically significant. Improvement was also observed in peak inspiratory flow and peak expiratory flow in the 6-month ribitol-treated group. However, significant improvement in limb muscle function was not demonstrated (Supplementary Figure 7b). Overall, these results showed that 5% ribitol oral treatment is able to enhance expression of F-α-DG with significant improvement in pathology of all muscles and in respiratory function.

### Early treatment with 10% ribitol improves muscle function

**Fig. 6:**
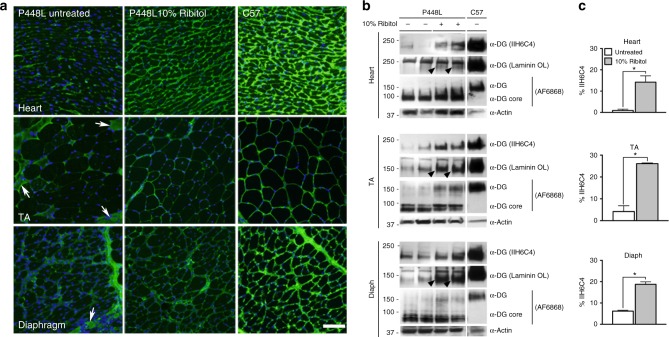
Induction of F-α-DG in cardiac and skeletal muscles of *P448L* mice treated with 10% ribitol from pregnancy. *P448L* breeding females were treated with 10% ribitol in drinking water at onset of pregnancy with pups continuing to receive treatment for 19 weeks. Untreated *P448L* mice were given drinking water only. **a** Immunohistochemical staining with IIH6C4 antibody of cardiac (heart), tibialis anterior (TA), and diaphragm muscles from the untreated and 10% ribitol-treated *P448L* mice (left and middle panel, respectively) and *C57* mice. Arrows indicate the degenerating fibers with staining for cytoplasmic Ig. Scale bar, 50 μm. Cellular nuclei were counterstained with DAPI (blue). **b** Western blot analysis of protein lysates from heart, TA and diaphragm (diaph) of two untreated (−) and two 10% ribitol-treated (+) *P448L*, and *C57* mice. F-α-DG was detected by blotting with IIH6C4 and by laminin overlay assay (Laminin OL). Core of α-DG was detected by AF6868 antibody. Detection of α-actin was used as a loading control. **c** Quantification of IIH6C4 band intensity from western blot. Values were normalized to α-actin expression for each tissue and presented as percentage expression compared to *C57*. Error bars represent mean ± SEM. Unpaired *t* test, *p ≤ 0.05

We reported recently that therapeutic outcome through restoration of F-α-DG depends on earlier treatment in the *P448L* mice^13,36^. Specifically, significant improvement in skeletal muscle functions by AAV gene therapy is achieved before the onset of the disease, but not in adult mutant mice when disease phenotype is already well established. To assess whether early intervention with a higher dose of ribitol can achieve significant improvement in skeletal muscle functions, we initiated a treatment with 10% ribitol in drinking water to the breeding females when they became pregnant and continued the treatment to the pups until they reached 19 weeks of age. All the functional tests were performed at the same age as the cohort treated with 5% ribitol from 7 week old for 3 months and age-matched untreated controls. Expression of F-α-DG was detected in all skeletal muscles and in the cardiac muscle of the 10% ribitol-treated mice by immunohistochemistry (Fig. 6a). F-α-DG was highly homogeneous in the cardiac muscle. Importantly, F-α-DG was clearly detected with even distribution in the skeletal muscles including the diaphragm. Expression of F-α-DG was clearly detected by western blots with the IIH6C4, reaching 14%, 18 and 26% normal levels in the heart, diaphragm and limb muscle, respectively (Fig. 6b, c). Enhanced expression of F-α-DG was also demonstrated by western blot with the antibody AF6868 (Fig. 6b). Finally, functionality of the ribitol-induced glycosylated α-DG was supported by laminin overlay assay (Fig. 6b).

**Fig. 7:**
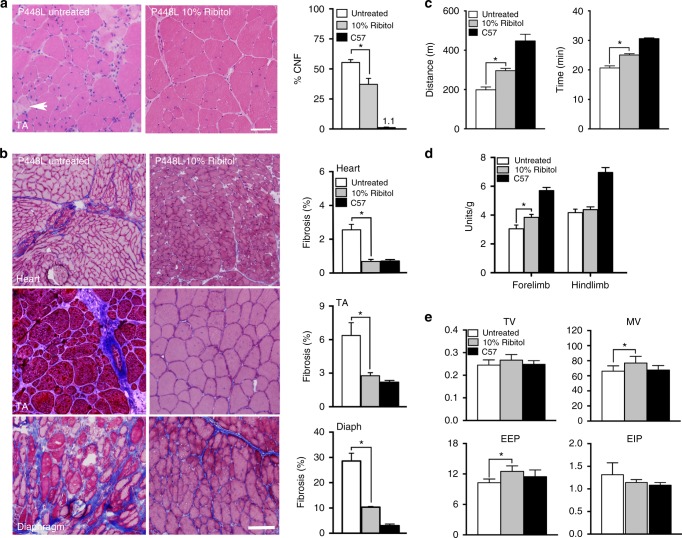
Effect of 10% ribitol treatment from pregnancy on histopathology and muscle function of *P448L* mice. Mice were treated from pregnancy to 19 weeks of age. Control *P448L* mice were given drinking water only. **a** H&E staining of tibialis anterior (TA) tissues from either untreated or 10% ribitol-treated *P448L* mice. Arrow indicates the degenerating fibers. Scale bar, 50 μm. Percentage of centrally nucleated fibers (% CNF) in TA muscles treated with 10% ribitol or aged-matched untreated *P448L* and *C57* mice (*n* = 3 for all cohorts). **b** Masson’s Trichrome staining of heart, TA (tibialis anterior), and diaphragm. Blue represents area of fibrosis. Percentage of fibrotic areas quantified from the treated and age-matched untreated *P448L* and *C57* (*n* = 3 for all cohorts) mice. **c** Treadmill exhaustion test assessing distance (m) and running time (min) in untreated (*n* = 10) or 10% ribitol-treated (*n* = 15) *P448L* mutant and *C57* mice (*n* = 10) at the age of 17 weeks. Unpaired *t* test, *p ≤ 0.05. **d** Grip strength test in untreated (*n* = 10) or 10% ribitol-treated (*n* = 15) *P448L* and *C57* mice (*n* = 10) at the age of 18 weeks. Force (Units) is normalized to body weight (g). **e** Respiratory function from untreated (*n* = 10) or 10% ribitol-treated (*n* = 15) *P448L* and *C57* control mice (*n* = 10) at 18 weeks of age (TV tidal volume, MV minute volume, EEP end-expiratory pause, EIP end-inspiratory pause). Error bars represent mean ± SEM. Unpaired *t* test, *p ≤ 0.05

Consistent with the enhancement on the biochemical marker, dystrophic pathology in the 10% ribitol-treated mice was greatly alleviated with significantly fewer CNFs (Fig. 7a). Most fibers of the limb muscles were highly homogenous in shape and size and only a proportion of fibers were centrally nucleated within the diseased muscles. Notably, improvement in pathology with reduced infiltration and fiber size variation was also observed in the diaphragm (Supplementary Figure 8). Furthermore, reduction in fibrosis was significant in cardiac muscle, and most prominent in the diaphragm (Fig. 7b).

Importantly, early treatment with 10% ribitol significantly improved skeletal muscle functions of the *P448*L mice. Treadmill tests showed that both running distance and time of the treated mice were significantly longer than the age-matched untreated mice (Fig. 7c). Grip strength tests also showed significant improvement on forelimb force from the ribitol-treated mice compared to the untreated (Fig. 7d). Significant improvement in respiratory functions were also demonstrated by plethysmography (Fig. 7e and Supplementary Figure 9a). Similar to the mutant mice treated for 6 months with 5% ribitol, a trend of improvement in TV, MV, end-expiratory and end-inspiratory pause (EEP and EIP, respectively) was observed, with MV and EEP reaching significant difference between the 10% ribitol-treated and the untreated *P448L* mice (Fig. 7e). Furthermore, improvement on expired volume (EV), relaxation time, and enhanced pause was also observed (Supplementary Figure 9a).

### Effects of ribitol on body weight and organ histology

No significant difference in body weight was observed between the mice treated with 10% ribitol from the embryonic stage, those treated with 5% ribitol from 7 weeks of age, or the age-matched untreated *P448L* mice at all time points, although treated female mice were slightly heavier than the controls (Supplementary Figure 9b).

The effect of ribitol treatment at the pharmacological concentration of 5% on histology of liver, kidney, and spleen was also evaluated with H&E staining. All tissues showed normal structure without degeneration and inflammation, and no difference was observed between the untreated and the ribitol-treated cohorts as illustrated in the Supplementary Figure 10a. We also performed biochemical analyses of serum markers for liver function including alkaline phosphatase, alanine transaminase, total bilirubin, conjugated bilirubin, and unconjugated bilirubin. In addition, kidney function was evaluated by analyses of urea (blood urea nitrogen) and creatinine levels. No statistical significance was observed between untreated and treated *P448L* mice. Levels of triglycerides and glucose were also similar between the two cohorts (Supplementary Figure 10b).

## Discussion

Despite significant advances in understanding the causes and clinical manifestation of dystroglycanopathies, almost no progress has been made for the treatment of the diseases including those caused by *FKRP* mutations. Currently, physical therapy and other clinic management routinely provided to patients only serve as palliative care. The only option of pharmacological intervention available is glucocorticoid steroids, which are being used anecdotally based on reported benefits from other muscular dystrophies, especially Duchenne muscular dystrophy^37^. Experimental therapy with the aim to restore F-α-DG by AAV-mediated gene therapy has been reported with high efficacy in preventing disease progression in mouse models^14^. However, clinical trials of the therapy for the diseases with such a wide range of phenotypes are challenging and remain to be conducted. Therefore, there is an urgent need for developing experimental therapies. Here we show that ribitol, a natural pentose alcohol present in some plants and animals and considered as a metabolic intermediate or end product, can effectively restore therapeutic levels of F*-*α-DG and, more importantly, ameliorate dystroglycanopathy caused by the *FKRP P448L* mutation, which is associated with severe CMD phenotype in clinic^38,39^. Our results offer a potentially safe and effective new class of treatment for restoration of F-α-DG to FKRP dystroglycanopathies. This treatment could be applied in combination with other therapies, such as AAV gene therapy for higher efficacy, by enhancing the function of FKRP transgene. The results also raise the potential of developing similar approaches for enhancing F-α-DG in cells of other diseases associated with aberrant *O*-mannosylation of α-DG. An example of such application is for cancers exhibiting reduced or lack of F-α-DG in association with invasion and metastasis, which can be inhibited by gene transfer-mediated upregulation of F-α-DG^5–7^.

FKRP dystroglycanopathy affects respiratory and cardiac muscles even in diseases with mild defects in skeletal muscles. Failures in respiratory and cardiac functions are the prime causes for the lethality of the diseases^38–41^. Therefore, restoration of F-α-DG and improvement in cardiac and respiratory functions are critically important for life quality and longevity of patients. Ribitol treatment enhances F-α-DG in both cardiac and diaphragm muscles, which is often most severely affected. This leads to significant improvements in the pathology of the diaphragm with striking reduction in fibrosis, which may explain the enhancement of respiratory functions. Cardiac defects in both pathology and functions in the *P448L* mice are limited and significant improvement in function is difficult to demonstrate even with effective AAV9 gene therapy^14^. Nevertheless, ribitol treatment is able to produce sustained and homogenous expression of F-α-DG in the treated cardiac muscle, resulting in significant reduction in fibrosis. All the data therefore clearly demonstrate therapeutic potential of the treatment to the two critical organs and their functions. Also important, ribitol treatment of different time frames up to 6 months shows no clear side effect. Oral ribitol administration from pregnancy to adult of the *P448L* mice does not affect pregnancy, embryo development, body weight, and overall behavior of the mutant mice. These together with normal histology and levels of serum markers for liver and kidney suggest the potential in safety for clinic applications.

**Fig. 8:**
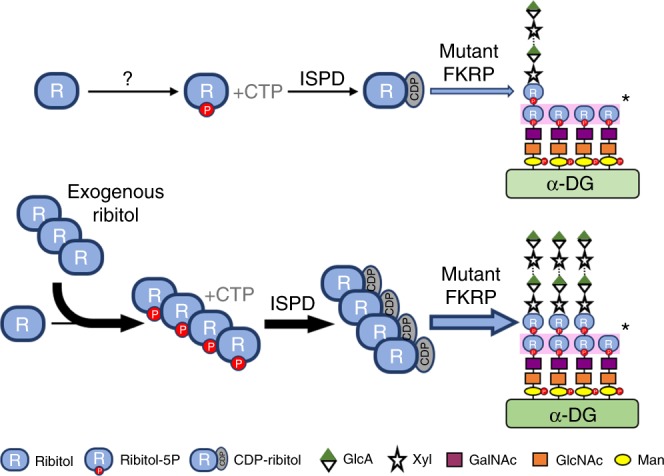
Model for ribitol-induced functional glycosylation of α-DG in FKRP mutant cells. “?” mechanism(s) is not understood, “*” first ribitol-5P on the Core M3 of α-DG is transferred by fukutin using also CDP-ribitol as the donor substrate

Therapeutic effects of ribitol treatment are related to the enhanced F-α-DG. Ribitol supplementation has been shown to increase the levels of CDP-ribitol both in cultured cells and in muscles of wild-type mice in vivo^23^. Our detection of increased levels of CDP-ribitol in both cardiac and skeletal muscles of *FKRP* mutant mice is consistent with the earlier report. We also demonstrated the increase in the levels of ribitol-5P and CDP-ribitol, which is considered the substrate of FKRP, indicating that ribitol can be effectively converted to CDP-ribitol in both FKRP mutant and normal muscle  tissues^23^. FKRP function is considered essential for functional glycosylation of α-DG. It is intelligible that the enhanced expression of F-α-DG in the diseased muscles also requires function of FKRP as in normal muscles. However, whether mutant FKRPs retain function has not been clearly determined till now. It has been demonstrated that diseased muscles with missense mutations of the *FKRP* gene remain capable of producing F-α-DG, but at variable lower levels (hypoglycosylation)^41–43^. This is most convincingly demonstrated in mouse models with *FKRP* mutations. The mutant mice with common *L276I* mutations express low but clearly detectable levels of F-α-DG in both skeletal and cardiac muscles. Considerable amount of F-α-DG is also detected in all compound heterozygotes containing *L276I* allele^33,34^. Despite the lack of expression of F-α-DG in the majority of muscle fibers of the *P448L* mice, individual muscle fibers are able to express near normal levels of F-α-DG with laminin-binding capacity. Normal levels of F-α-DG is expressed in all regenerating fibers^44^. However, direct evidence came only recently from AAV-mediated expression of the P448L mutant FKRP, which is able to restore F-α-DG and protect muscles from degeneration^32^. Interestingly, strong expression of F-α-DG is detected in all newborn skeletal and cardiac muscles of the *P448L* mice and this expression is not associated with increase in mutant FKRP expression^45^. This demonstration supports the functionality of the mutant FKRPs and, more importantly, suggests that factor(s) other than FKRP expression could compensate for the reduced functionality of the mutant FKRPs. We therefore hypothesize that the additional amount of ribitol allows the muscle fibers to produce higher than normal levels of FKRP substrate (CDP-ribitol), which enhances and partially compensates for the reduced function of mutant FKRPs (Fig. 8). Fortunately, more than 90% of patients with *FKRP* mutations contain at least one allele of the *L276I* mutation, which retain at least partial function as demonstrated in patient muscles and in the mutant mouse models. Therefore, this low cost and easy to administer experimental therapy will likely be applicable to the majority of patients with *FRKP* mutations^38,46,47^. This together with the nature of the drug, expected to be of limited side effects, would make execution of an early clinical trial much simpler. Further studies to understand the pharmacokinetics of ribitol in disease models and metabolic pathways of the pentose alcohol to CDP-ribitol would also allow us to optimize the dosage and delivery regime for higher efficacy. Since details of the mammalian ribitol-5P biosynthetic pathway remains unknown, potential non-glycosylation-related effect of pharmacological dose of ribitol on muscles and other systems requires attention.

## Methods

### Animal care

All animal studies were approved by the Institutional Animal Care and Use Committee of Carolinas Medical Center. All mice were housed in the vivarium of Carolinas Medical Center following animal care guidelines of the institute. Animals were ear tagged prior to group assignment. Food and water were available ad libitum during all phase of the study. Body weight was measured from 6 weeks to 19 weeks of age.

### Mouse model and experimental procedure

*FKRP P448L* mutant mice were generated by the McColl-Lockwood Laboratory for Muscular Dystrophy Research^32,33^. The mice contain a homozygous missense mutation (*c.1343C>T*, p.Pro448Leu) in the *FKRP* gene with the floxed neomycin-resistant (Neo^r^) cassette removed from the insertion site. *C57BL/6* (wild-type/C57) mice were purchased from the Jackson Laboratory.

Ribitol was purchased from Sigma (A5502 Adonitol, >98%, Sigma, St. Louis, MO, USA) and dissolved in drinking water to the final concentration of 5% or 10%. *P448L* mice aged at 4 weeks were treated with 5% ribitol drinking water for 1 month and *P448L* mice aged at 7 weeks were treated with 5% ribitol drinking water for 3 and 6 months. All the mice were randomly assigned to either treatment or control groups. A minimum number of four mice was used for each group. No animal was excluded. *P448L* female breeders were given 10% ribitol in drinking water during pregnancy, and pups continued to be treated with 10% ribitol in drinking water until they were euthanized at 19 weeks of age. Untreated age-matched *P448L* and wild-type *C57BL/6* mice were used as controls. The animals were terminated at the end of each treatment time point and tissues including heart, diaphragm, TA, quadriceps, liver, spleen, and kidney were collected for analyses.

### Immunohistochemical and western blot analysis

Tissues were dissected and snap-frozen in dry ice-chilled 2-methylbutane. For immunohistochemical detection of functionally glycosylated α-DG, 6-μm-thick cross-sections of untreated and *C57* control, as well as tissues from treated cohorts were included in each slide. Slides were first fixed in ice-cold ethanol:acetic acid (1:1) for 1 min, blocked with 10% normal goat serum (NGS) in 1× Tris-buffer saline (TBS) for 30 min at room temperature, and incubated overnight at 4 °C with primary mouse monoclonal antibody IIH6C4 (EMD Millipore, 05-593, 1:500) against α-DG. Negative controls received 10% NGS in 1× TBS only. Sections were washed and incubated with secondary AlexaFluor 488 goat anti-mouse IgM (Invitrogen, A-21042, 1:500) at room temperature for 2 h. Sections were washed and finally mounted with fluorescence mounting medium (Dako) containing 1× DAPI (4′,6′-diamidino-2-phenylindole) for nuclear staining. Immunofluorescence was visualized using an Olympus BX51/BX52 fluorescence microscope (Opelco) and images were captured using the Olympus DP70 digital camera system (Opelco). Slides were examined in a blinded manner by the investigator.

For western blot analysis, tissues were homogenized in the extraction buffer (50 mM Tris-HCl, pH 8.0, 150 mM NaCl, and 1% Triton X-100), and then supplemented with 1× protease inhibitor cocktail (Sigma-Aldrich). Protein concentration was quantified by the Bradford assay (Bio-Rad DC protein assay). Eighty micrograms of protein was loaded on an 4–15% Bio-Rad Mini-PROTEAN TGX gel (Bio-Rad) and immunoblotted. Amount of total protein loaded for *C57* mice was half of the amount loaded for the *P448L* mice. Nitrocellulose membranes (Bio-Rad) were blocked with 5% milk in 1× phosphate-buffered saline for 2 h at room temperature and then incubated with the following primary antibodies overnight at 4 °C: IIH6C4 (1:2000), AF6868 (R&D Systems, AF6868, 1:1000), and α-actin (Sigma, SAB4502543, 1:1000). Appropriate horseradish peroxidase (HRP)-conjugated secondary antibodies were incubated for 2 h at room temperature. All blots were developed by electrochemiluminescence immunodetection (PerkinElmer). For IIH6C4 band quantification from western blot ImageJ software was used. For laminin overlay assay, nitrocellulose membranes were blocked with laminin overlay buffer (10 mM ethanolamine, 140 mM NaCl, 1 mM MgCl_2_, and 1 mM CaCl_2_, pH 7.4) containing 5% nonfat dry milk for 1 h at 4 °C, followed by incubation with laminin from Engelbreth–Holm–Swarm murine sarcoma basement membrane (Sigma, L2020) at a concentration of 2 μg/ml overnight at 4 °C in laminin overlay buffer. Membranes were then incubated with rabbit anti-laminin antibody (Sigma, L9393,1:1500), followed by goat anti-rabbit HRP-conjugated immunoglobulin G secondary antibody (Santa Cruz Biotechnology, sc-2030, 1:3000). Blots were saturated with Western-Lightning Plus ECL (PerkinElmer) before exposure to and developing of GeneMate auto-radiographic film (VWR). The uncropped scans of western blots for the detection of glycosylated α-DG are provided as Supplementary Figure 11 within the Supplementary Information.

### Histopathological and morphometric analysis

Frozen tissues were processed for H&E and Masson's Trichrome staining following standard procedures. Muscle cross-sectional fiber equivalent diameter was determined from tibialis anterior and quadriceps stained with H&E using the MetaMorph v7.7 Software (Molecular Devices). The percentage of centrally nucleated myofibers were manually quantified from the same tissue sections stained with H&E. Fibrotic area represented by blue staining in the Masson’s Trichrome-stained sections was quantified from heart, diaphragm, tibialis anterior, and quadriceps using the ImageJ software. For all the morphometric analyses, a total of 300 to 400 fibers from two representative ×20 magnification images per each muscle per animal was used.

### Quantitative reverse transcriptase PCR assay

Tissues were collected from heart, diaphragm, and tibialis anterior. RNA was extracted using TRIzol (Invitrogen) following the supplied protocol. Final RNA pellet was re-suspended in 20 μl RNAse-nuclease-free water. Final RNA concentration was determined using Nanodrop 2000c. One microgram of RNA was subsequently converted to complementary DNA (cDNA) using the High-Capacity RNA-to-cDNA™ Kit (Applied Biosystems) following the supplied protocol. cDNA was then used for quantitative real-time PCR using the mouse FKRP-FAM (Mm00557870_m1) and LARGE-FAM (Mm00521885_m1) TaqMan gene expression assay with primer limited GAPDH-VIC (Mm99999915_g1) as the internal control (Thermo Fisher Scientific) and TaqMan^®^ Universal Master Mix II, with UNG (Life Technologies). Quantitative real-time PCR was run on the Bio-Rad CFX96 Touch™ Real-Time PCR Detection System (Bio-Rad) following the standard real-time PCR conditions suggested for TaqMan assays. Results of FKRP and LARGE transcript were calculated and expressed as 2^−∆∆Ct^ and compared across tissues and animals.

### Metabolite extraction and LC/MS-MS analysis

Ribitol was purchased from Sigma (A5502). Ribitol-5P and CDP-ribitol were synthesized by Z-Biotech (Aurora, CO, USA). Muscle tissues were collected, and blinded samples were subjected to the following procedure. Thirty to eighty micrograms of frozen tissue samples were homogenized with 400 μl of MeOH:acetonitrile (ACN) (1:1) and then centrifuged for 5 min at 8000 × *g*. The supernatants were removed, transferred to individual wells of 96-well plate, and analyzed by LC/MS-MS. An Applied Biosystems Sciex 4000 (Applied Biosystems, Foster City, CA, USA) equipped with a Shimadzu HPLC (Shimadzu Scientific Instruments Inc., Columbia, MD, USA) and Leap auto-sampler (LEAP Technologies, Carrboro, NC, USA) were used to detect ribitol, ribitol-5P, and CDP-ribitol from tissue samples and synthetic compounds. The metabolites were separated on a silica gel column (Hypersil Silica 250 × 4.6 mm^2^, 5 μm particle size) using solvent A: water, 10 mM NH_4_OAc, 0.1% formic acid, and solvent B: MeOH:ACN (1:1). The following gradient was used: 0–12 min, 5% buffer B; 13–14 min, 95% buffer B, and 15–17 min, 5% buffer B. Under these conditions, ribitol, ribitol-5P, and CDP-ribitol eluted at 8.3, 7.5, and 8.9 min, respectively. The metabolites were analyzed using electrospray ionization mass spectrometry operated in positive ion mode, ESI+. Compounds concentration in tissue samples were determined based on standard curves prepared by serial dilutions (200–0.01 μM) of each of the compound in MeOH:ACN (1:1).

### Cell culture and LC/MS-MS analysis of labeled metabolites

C2C12 mouse myoblast (ATCC, CRL-1772) were seeded and grown in Dulbecco's modified Eagle's medium (DMEM) GlutaMax medium (Gibco by Life Technologies) and then supplemented with 10% fetal bovine serum and 100 μg/ml penicillin–streptomycin. Differentiation into myotubes was induced by replacing the growth media with DMEM supplemented with 1 μM insulin (Sigma), 2% heat-inactivated horse serum (Gibco by Life Technologies), 2.5 μM dexamethasone (Sigma), and 5 mM ^13^C-ribitol (Omicron Biochemicals Inc., South Bend, IN, USA) when cells reached confluence. Cells were harvested 5 days later and analyzed by LC/MS-MS for detection of ribitol (153.2 → 98.8*m*/*z*), ribitol-5P (233.1 → 98.8*m*/*z*), CDP-ribitol (538.1 → 324.1*m*/*z*), ^13^C-ribitol (158.3 → 103.8*m*/*z*), ^13^C-ribitol-5P (238.1. → 98.8*m*/*z*), and CDP-^13^C-ribitol (543.1. → 324.1*m*/*z*) as described above.

### Muscle function tests

For treadmill exhaustion test, 17- and 30-week-old mice were placed on the belt of a five-lane-motorized treadmill (LE8700 treadmill, Panlab/Harvard Apparatus, Barcelona, Spain) supplied with shock grids mounted at the back of the treadmill, which delivered a 0.2 mA current to provide motivation for exercise. Initially, the mice were subjected to an acclimation period (time, 5 min; speed, 8 cm/s, and 0° incline). Immediately after acclimation period, the test commenced with speed increases of 2 cm/s every minute until exhaustion. The test was stopped and the time to exhaustion was determined when the mouse remained on the shock grid for 5 s without attempting to re-engage the treadmill^14^.

For grip force test, forelimb and hindlimb in peak torque was measured by a grip strength meter (Columbus Instruments). For forelimb force, the animal was held so that only the forelimb paws grasp the specially designed mouse flat mesh assembly, and was pulled back from the tail until the grip was broken. The force transducer recorded the peak force reached when the animal’s grip is broken. For hindlimb force, an angled mesh assembly was used. Mice were allowed to rest on the angled mesh assembly, facing away from the meter with its hindlimbs at least one-half of the way down the length of the mesh. The mouse tail was pulled directly toward the meter and parallel to the mesh assembly. During this procedure, the mice resist by grasping the mesh with all four limbs. Pulling toward the meter was continued until the hindlimbs released from the mesh assembly. Five successful hindlimb and forelimb force measurements within 2 min were recorded. The average value was used for analysis. Force was presented as values of KGF (kilogram-force) units normalized to bodyweights (g) as “Units/g”. The tests were performed 1 week before euthanasia.

### Whole-body plethysmography

Respiratory functional analysis in conscious, freely moving 18- and 31-week-old mice were measured using a whole-body plethysmography technique. The plethysmograph apparatus (emka Technologies, Falls Church, VA, USA) was connected to a ventilation pump for the purpose of maintaining a constant air flow, a differential pressure transducer, a usbAMP signal amplifier, and a computer running EMKA iox2 software with the respiratory flow analyzer module, which was used to detect pressure changes due to breathing and recording the transducer signal. An initial amount of 20 ml of air was injected and withdrawn via a 20 ml syringe into the chamber for the purpose of calibration. Mice were placed inside the free-moving plethysmograph chamber and allowed to acclimate for 5 min in order to minimize any effects of stress-related changes in ventilation. Resting ventilation was measured for a duration of 15 min after the acclimation period. Body temperatures of all mice were assumed to be 37 °C and to remain constant during the ventilation protocol^14^.

### Statistical analysis

All data are expressed as mean ± SEM unless stated otherwise. Statistical analyses were performed with GraphPad Prism version 7.01 for Windows (GraphPad Software). Individual means were compared using multiple *t* tests. Differences were considered to be statistically significant at *p* ≤ 0.05 (*).

### Data availability

The authors declare that all data supporting the findings of this study are available within the article and its Supplementary Information Files.

## Electronic supplementary material

Supplementary Information
