# Supplementary material for: Ribitol restores functionally glycosylated α-dystroglycan and improves muscle function in dystrophic FKRP-mutant mice
Source: Nat Commun. 2018 Aug 27;9:3448. doi: 10.1038/s41467-018-05990-z (PMC6110760; doi:10.1038/s41467-018-05990-z)
Supplement: Supplementary file 1 — Supplementary Information [file 41467_2018_5990_MOESM1_ESM.pdf]

Supplementary Information

Ribitol restores functionally glycosylated α-dystroglycan and improves muscle functions in dystrophic *FKRP*-mutant mice

Cataldi et al.

Supplementary Figures

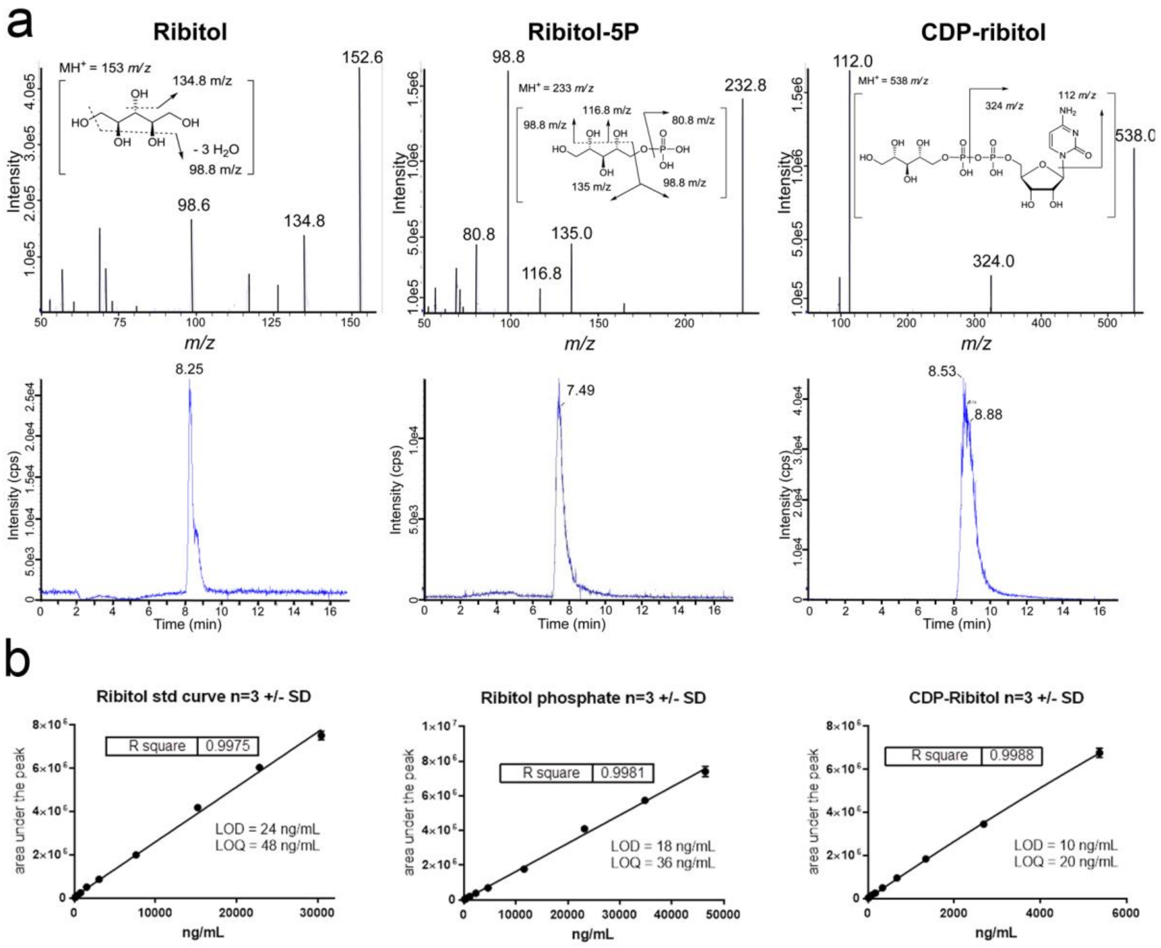

Supplementary Figure 1. LC/MS-MS chromatograms for the detection and quantification of synthetic ribitol, ribitol-5P and CDP-ribitol. (**a**) MS-MS Spectrum (upper panels) and chromatograms with retention time (lower panels) of the synthetic ribitol, ribitol-5P and CDP-ribitol. **(b)** Standard curve from serial dilution of stock solutions for each metabolite.

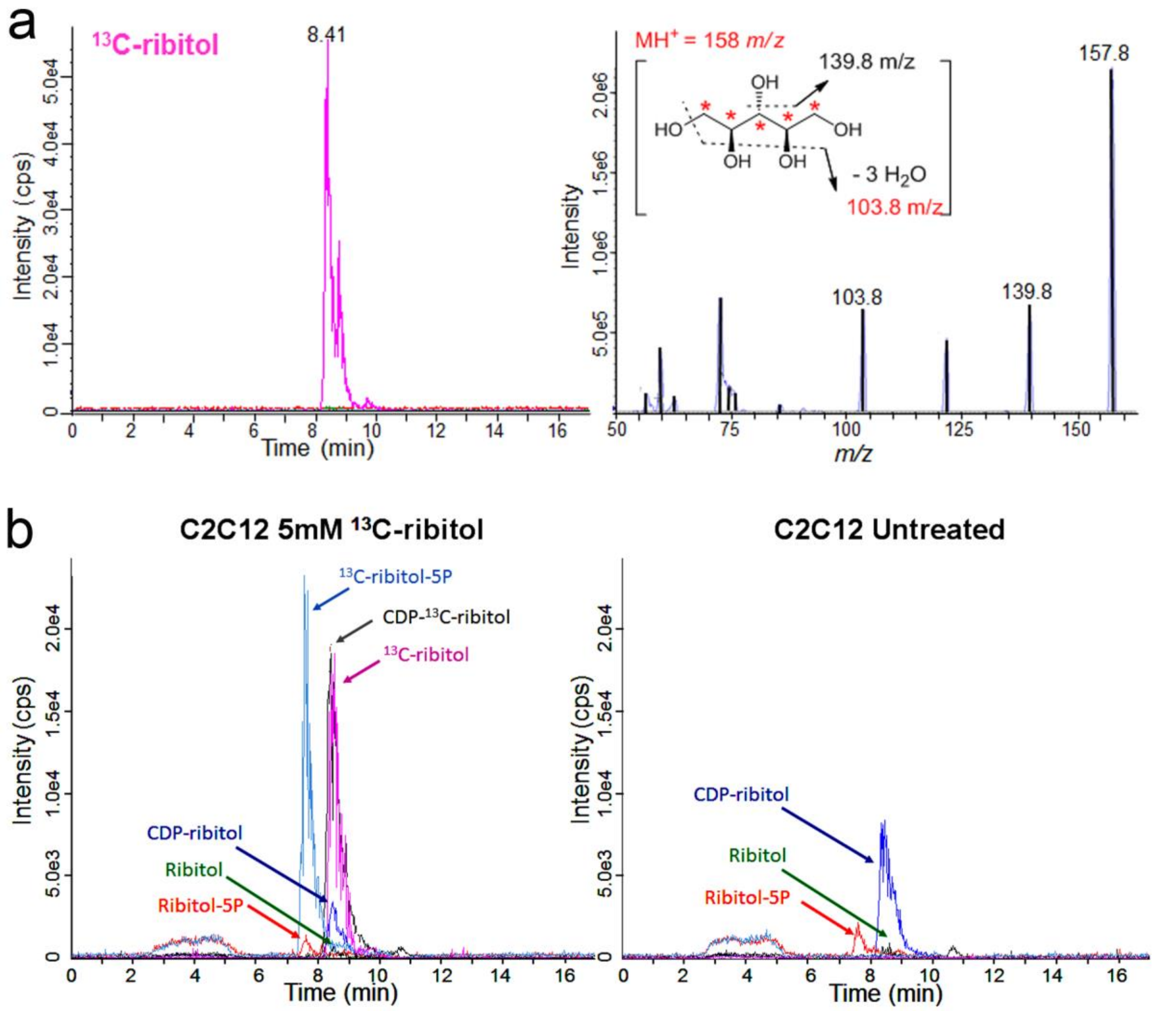

Supplementary Figure 2. LC/MS-MS chromatograms for the detection of isotopically labeled ^{13}C-ribitol, ^{13}C-ribitol-5P and CDP-^{13}C-ribitol. (**a**) ^{13}C-ribitol chromatogram with retention time and MS-MS Spectrum with fragmentation. **(b)** LC/MS-MS detection of ^{13}C-ribitol, ^{13}C-ribitol-5P, CDP-^{13}C-ribitol, and their unlabeled analogs from untreated and 5mM ^{13}C-ribitol-treated differentiated C2C12 myotubes *in vitro*.

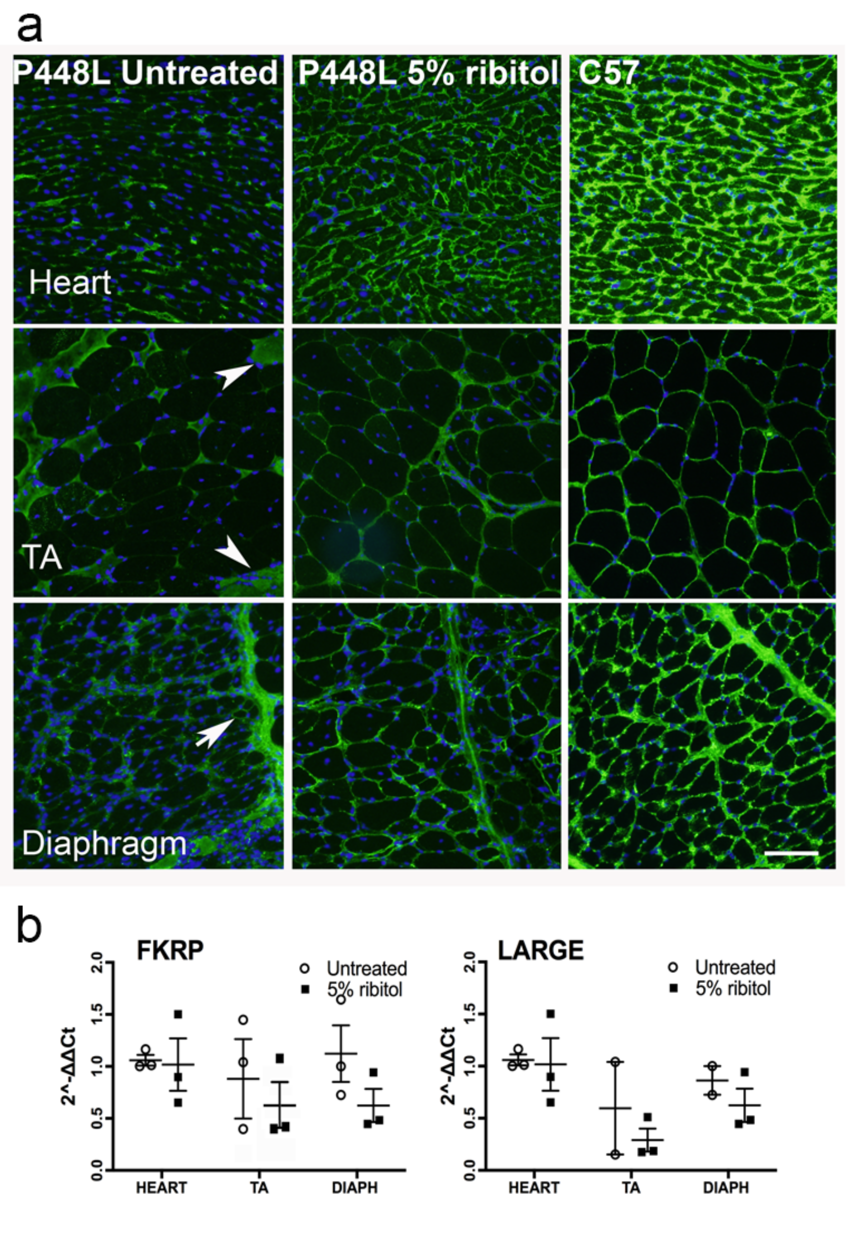

Supplementary Figure 3. Induction of F-α-DG in three-month 5% ribitol-treated *P448L* mutant mice. (**a**) Immunohistochemical staining for F-α-DG with IIH6C4 antibody in cardiac (heart), tibialis anterior (TA), and diaphragm muscles from *P448L* mice drinking either water (P448L untreated) or water supplemented with 5% ribitol (P448L 5% ribitol) and wild-type *C57* mice. Arrow indicates the revertant fibers expressing detectable F-α-DG and arrow heads

indicate the degenerating fibers. Cellular nuclei were counterstained with DAPI (blue). Scale bar, 50 μm. (**b**) Levels of FKRP and LARGE transcripts in cardiac muscle (heart), skeletal muscle (tibialis anterior, TA) and diaphragm (diaph) analyzed by quantitative real-time PCR (n=3). Error bars represent mean ± SEM. Unpaired t test, * $p<0.05$.

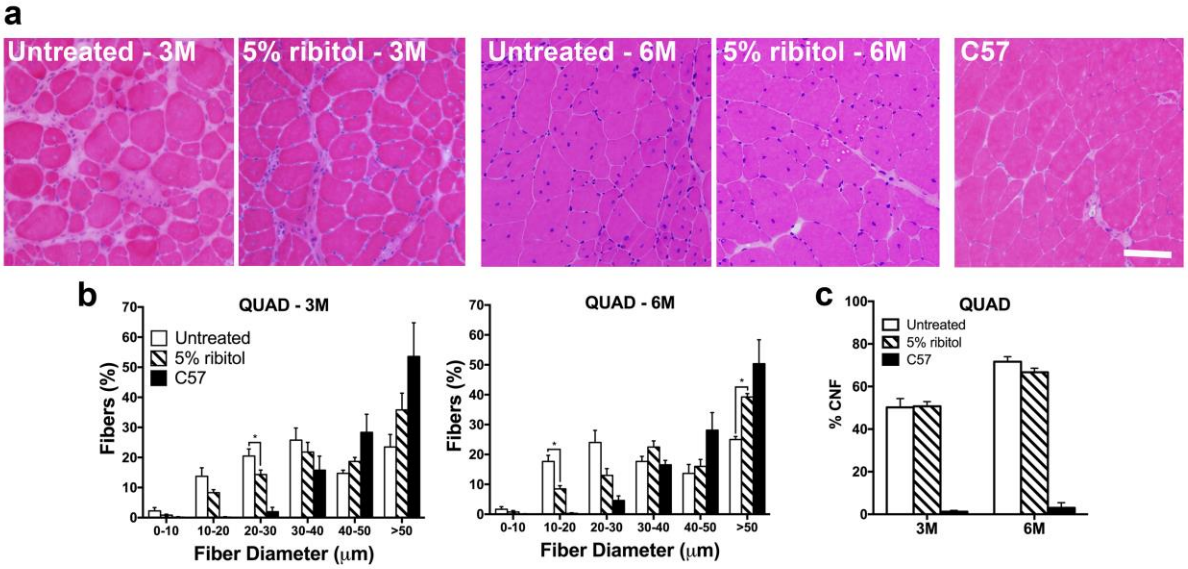

Supplementary Figure 4. Effect of 5% ribitol treatment on histopathology of *P448L* mutant mice. (**a**) H&E staining of quadriceps from *P448L* mutant mice drinking either water (Untreated) or water supplemented with 5% ribitol (5% ribitol). Treatments were maintained for either 3 (3M) or 6 months (6M). Scale bar, 50 μm. (**b**) Fiber size distribution from quadriceps of either 5% ribitol treated (n=6) or age-matched untreated (n=3) *P448L* mutant mice, and wild-type *C57* (n=3) mice. (**c**) Percentage of centrally-nucleated fibers (% CNF) from quadriceps of 3M (n=6) and 6M (n=4) ribitol-treated or age-matched untreated (n=3) *P448L* mutant mice, and wild-type *C57* mice (n=3). Error bars represent mean ± SEM. Unpaired t test *p<0.05.

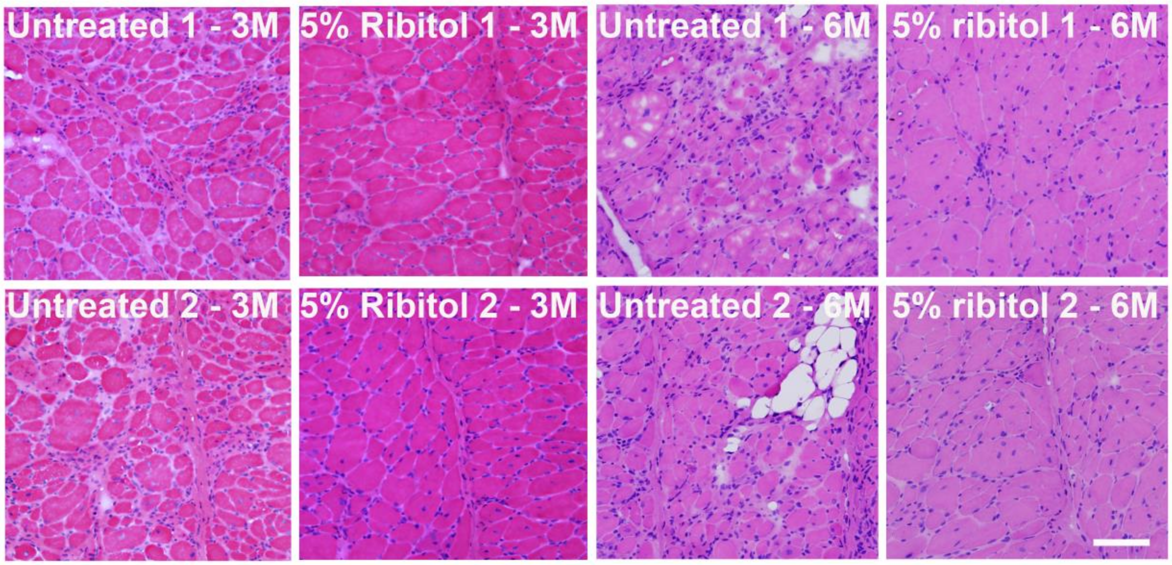

Supplementary Figure 5. Histopathology of diaphragms from 5% ribitol-treated *P448L* mutant and control mice. H&E staining of diaphragms from two untreated (Untreated 1 and 2) and two 5% ribitol-treated (5% Ribitol 1 and 2) *P448L* mutant mice. Treatments were maintained for either 3 months (3M) or 6 months (6M). Scale bar, 50 μm.

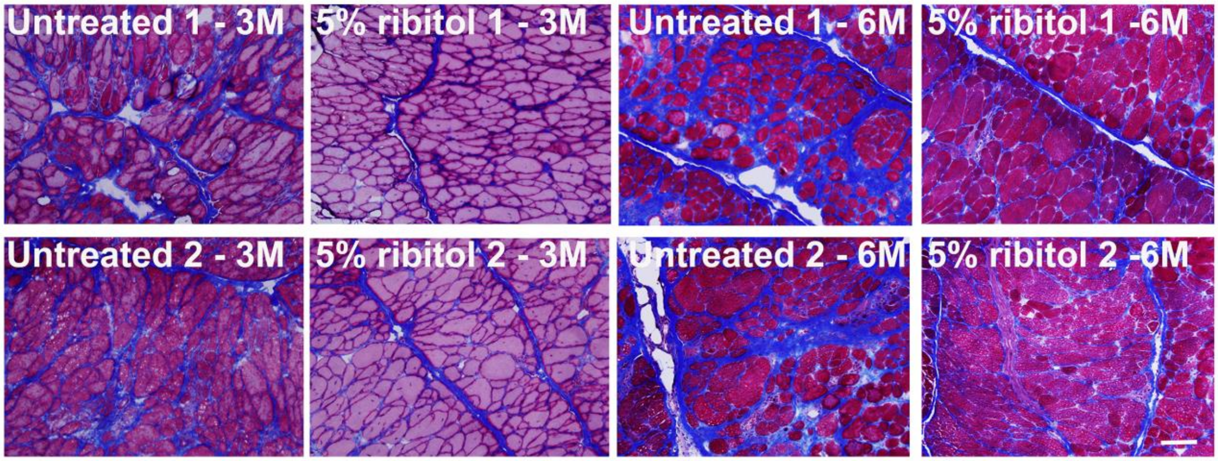

Supplementary Figure 6. Fibrosis in diaphragms of untreated and 5% ribitol-treated *P448L* mutant mice. Masson's Trichrome staining of diaphragms from two untreated (Untreated 1 and 2) and two 5% ribitol-treated (5% ribitol 1 and 2) *P448L* mutant mice. Treatments were maintained for either 3 months (3M) or 6 months (6M). Scale bar, 50 μm.

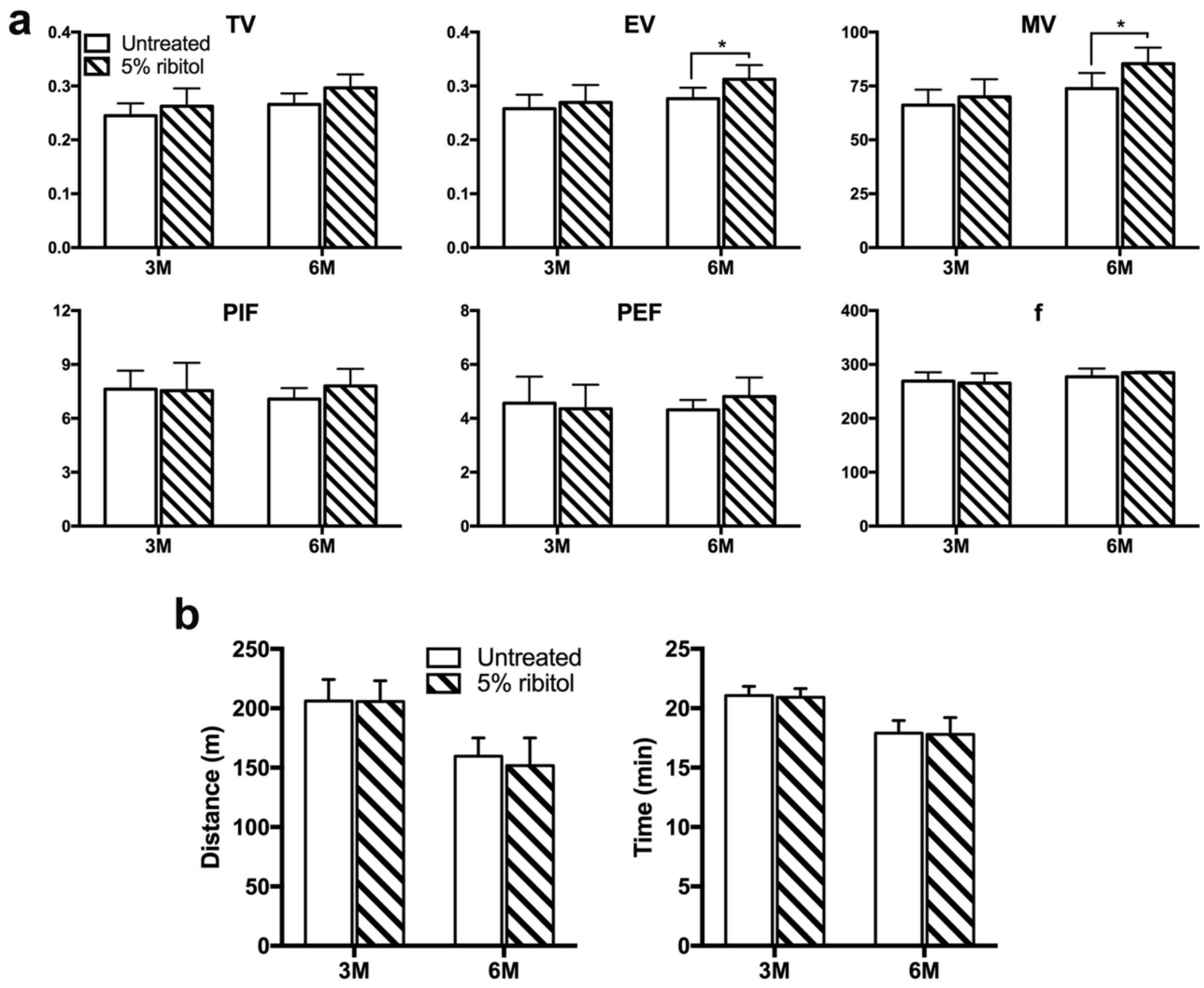

Supplementary Figure 7. Evaluation of respiratory skeletal muscle function in 5% ribitol-treated *P448L mutant* mice. Seven-week-old *P448L* mutant mice were given drinking water only, or drinking water supplemented with 5% ribitol for either 3 months (3M) or 6 months (6M). (**a**)**.** Respiratory function parameters from untreated (n=10 for 3M and 6M) or 5% ribitol-treated (n=10 for 3M, n=4 for 6M) *P448L* mice. (TV: tidal volume, EV: expiratory volume, MV: minute volume, PIF: peak inspiratory flow, PEF: peak expiratory flow, and f: breathing frequency). (**b**). Treadmill exhaustion test assessing the distance (m, meters) and time (min, minutes) until exhaustion run by untreated (n=10 for 3M and 6M) or 5% ribitol-treated (n=10 for 3M, n=4 for 6M) *P448L* mutant mice. Error bars represent mean ± SEM. Unpaired t test *$p<0.05$

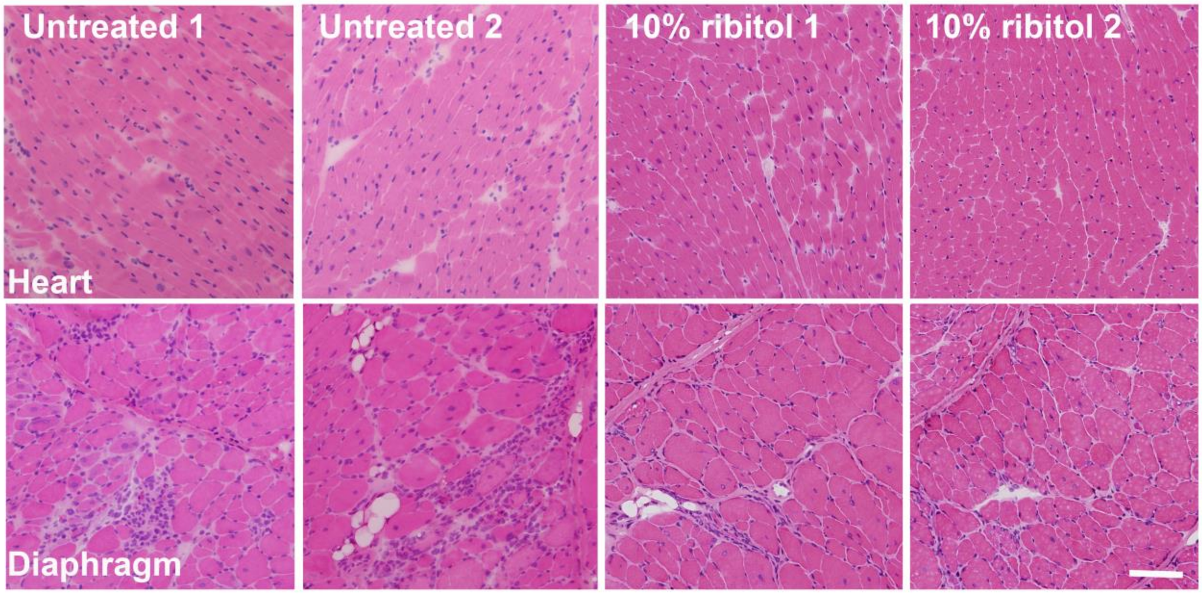

Supplementary Figure 8. Histopathology in skeletal and cardiac muscles of *P448L* mutant mice treated with 10% ribitol. *P448L* mutant mice were treated with 10% ribitol in drinking water when the breeding female became pregnant, and the pups continued to be treated until 19 weeks of age. Untreated *P448L* mutant mice were given drinking water only. H&E staining of heart and diaphragm tissues from either untreated (Untreated 1 and 2), or 10% ribitol-treated (10% ribitol 1 and 2) *P448L* mutant mice. Scale bar, 50 μm.

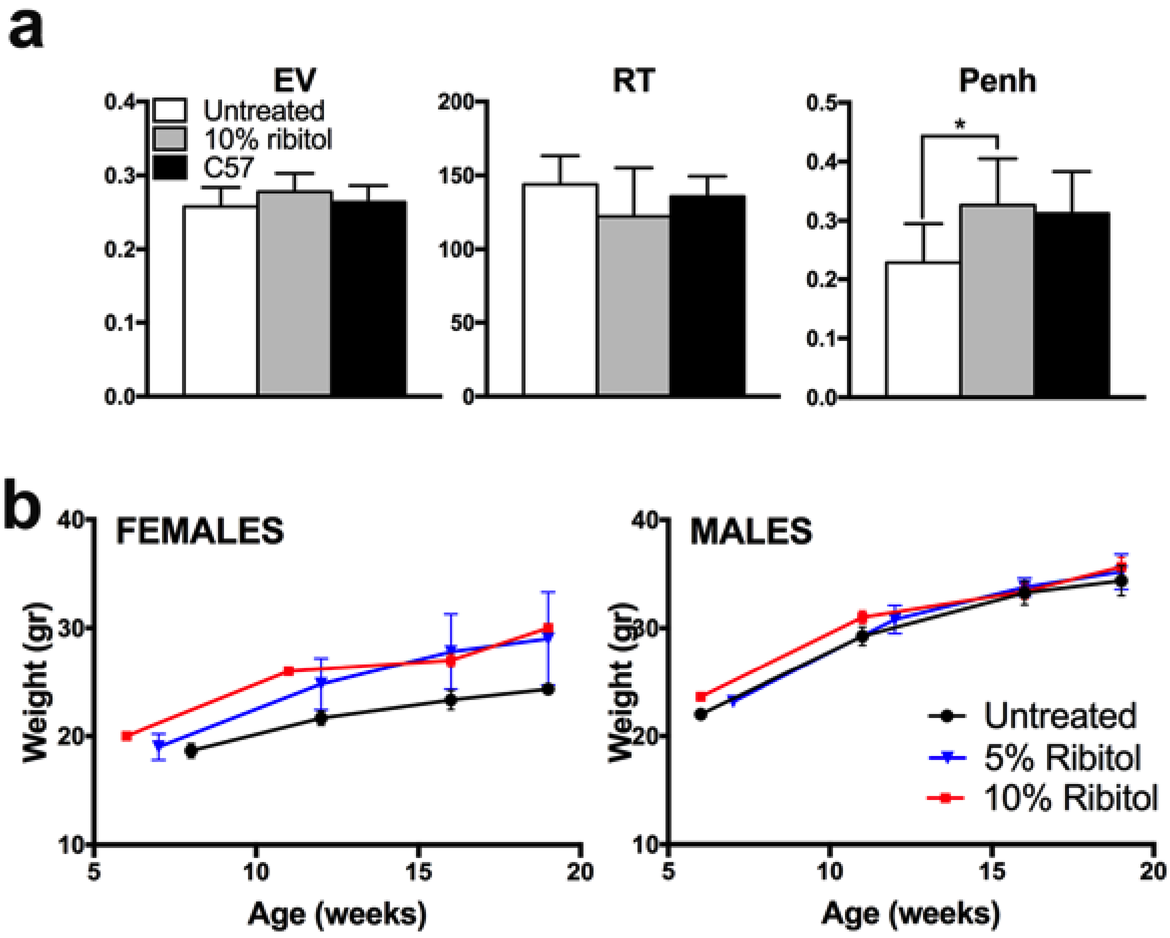

Supplementary Figure 9. Effect of 10% ribitol treatment on respiratory function and body weight of *P448L* mutant mice. *P448L* mutant mice were treated with 10% ribitol in drinking water when the breeding female became pregnant, and the pups continued to be treated until 19 weeks of age. Untreated *P448L* mutant mice were given drinking water only. (**a**) Respiratory function parameters from untreated (n=10) or 10% ribitol-treated (n=15) *P448L* mice, and C57 control mice at 18 weeks of age. (EV: expiratory volume, RT: relaxation time, Penh: enhanced pause). Error bars represent mean ± SEM. Unpaired t test *p<0.05. (**b**) Body weight (gr) change among the mice treated from the embryonic stage (10% ribitol) or those treated from 7 weeks of age (5% ribitol) in comparison with age-matched untreated *P448L* mutant mice.

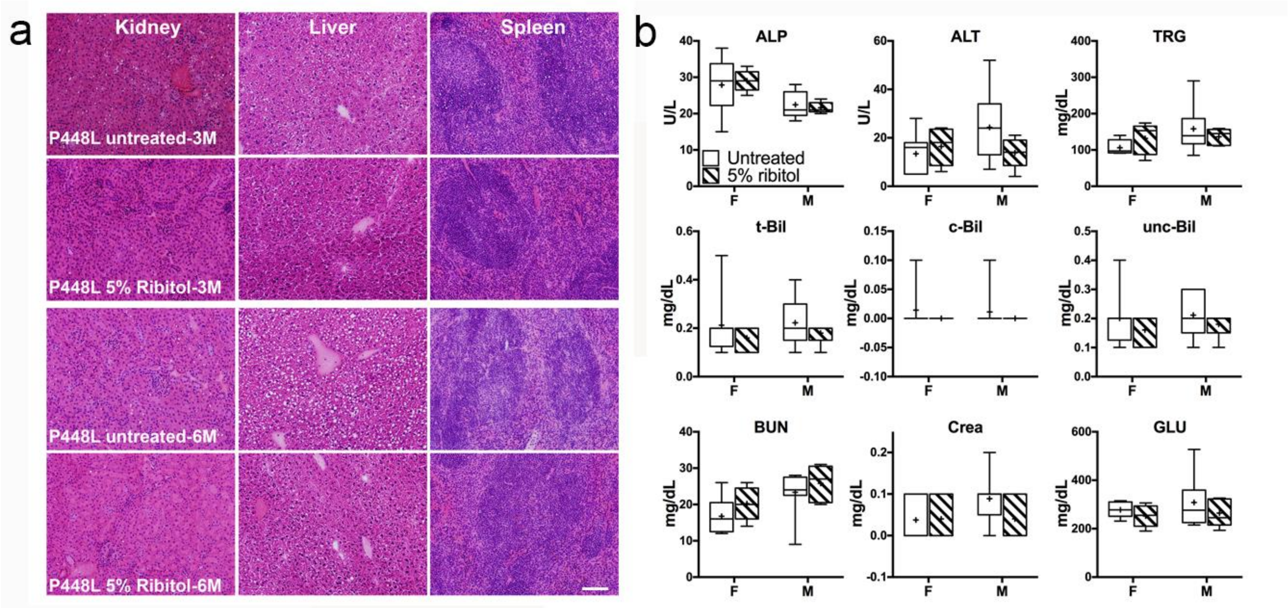

Supplementary Figure 10. Evaluation of ribitol toxicity in kidney, liver, spleen and serum. **(a)** H&E staining of kidney, liver and spleen from *P448L* mice drinking water only (untreated) or water supplemented with 5% ribitol (5% ribitol). Treatment were maintained for 3 (3M) or 6 months (6M). Scale bar, 100 μm **(b)** Levels of serum biochemical analytes from *P448L* females (F) and males (M) mice, either untreated or treated with 5% ribitol for 6 months. (Untreated F, n=8; untreated M, n=9; treated F, n=5; treated M, n=5). (ALP; alkaline phosphatase, ALT: alanine transaminase, TRG: triglycerides, t-Bill: total bilirubin, c-Bil: conjugated bilirubin, unc-Bil: unconjugated bilirubin, BUN: urea, Crea: creatinine, GLU: glucose). Box represents 25th and 75th percentiles. Line represents median. “+” represents mean. Whiskers extend from minimum to maximum value.

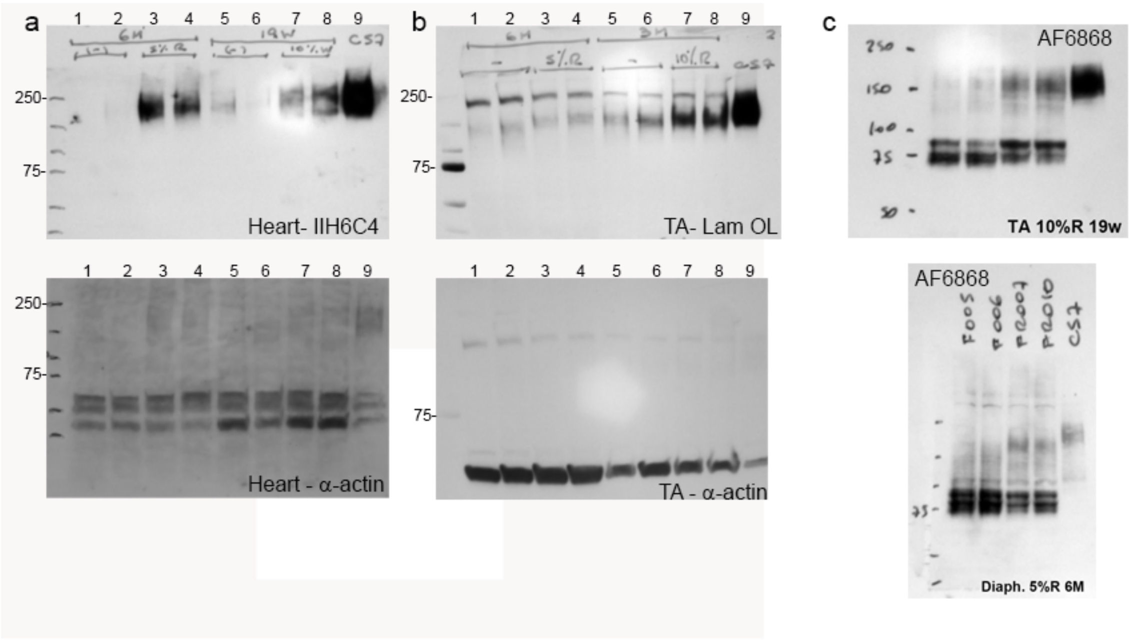

Supplementary Figure 11. Uncropped Western Blots and laminin overlay assays. (**a**) Uncropped images corresponding to western blot from heart tissue stained with IIH6C4 (upper) and α-actin (lower) antibodies presented in Fig. 3 (wells 1 to 4, and 9), and Fig. 6 (wells 5 to 9). (**b**) Upper panel: Uncropped images corresponding to laminin overlay assay (Lam OL) from tibialis anterior (TA) muscle presented in Fig. 3 (wells 1 to 4, and 9), and Fig. 6 (wells 5 to 9). Lower panel: Uncropped images corresponding to western blot from TA muscle stained with α-actin antibody presented in Fig. 3 (wells 1 to 4, and 9), and Fig. 6 (wells 5 to 9). (**c**) Uncropped images corresponding to western blots stained with AF6868 antibody from tibialis anterior tissue presented in Fig. 6 (upper panel), and diaphragm presented in Fig. 3 (lower panel).
